# Targeting Cytokines as Evolving Treatment Strategies in Chronic Inflammatory Airway Diseases

**DOI:** 10.3390/ijms19113402

**Published:** 2018-10-30

**Authors:** Jaleesa Garth, Jarrod W. Barnes, Stefanie Krick

**Affiliations:** Division of Pulmonary, Allergy and Critical Care Medicine, Department of Medicine, The University of Alabama at Birmingham, Birmingham, AL 35294, USA; jmgarth6@uab.edu (J.G.); jbarnes@uabmc.edu (J.W.B.)

**Keywords:** COPD, allergic asthma, bronchiectasis, cytokines, IL-1β, IL-6, IL-8

## Abstract

Cytokines are key players in the initiation and propagation of inflammation in chronic inflammatory airway diseases such as chronic obstructive pulmonary disease (COPD), bronchiectasis and allergic asthma. This makes them attractive targets for specific novel anti-inflammatory treatment strategies. Recently, both interleukin-1 (IL-1) and IL-6 have been associated with negative health outcomes, mortality and a pro-inflammatory phenotype in COPD. IL-6 in COPD was shown to correlate negatively with lung function, and IL-1beta was induced by cigarette smoke in the bronchial epithelium, causing airway inflammation. Furthermore, IL-8 has been shown to be a pro-inflammatory marker in bronchiectasis, COPD and allergic asthma. Clinical trials using specific cytokine blockade therapies are currently emerging and have contributed to reduce exacerbations and steroid use in COPD. Here, we present a review of the current understanding of the roles of cytokines in the pathophysiology of chronic inflammatory airway diseases. Furthermore, outcomes of clinical trials in cytokine blockade as novel treatment strategies for selected patient populations with those diseases will be discussed.

## 1. Introduction

Chronic respiratory diseases account for 4.7% of global disability-adjusted life years with asthma and chronic obstructive pulmonary disease (COPD) being the most frequent, and chronic airway inflammation being one of the key processes involved in their pathogenesis [1,2].

Since the lung is constantly exposed to the environment and harmful pathogens, inflammation, as an immediate defense mechanism, is required to eliminate these noxious stimuli as early as possible. For a long time, inflammatory cells were thought to be the key orchestrators of the immune homeostasis in the lung, with dendritic cells as antigen-presenting cells and macrophages as the first line of defense. Macrophages are located in airways, alveoli and the lung interstitium, and have the ability to migrate to the lung microvasculature, thereby modulating acute and chronic inflammatory responses [3]. They are also a main source of cytokines and inflammatory mediators, in addition to phagocytosing bacteria and apoptotic cells, and promote neutrophil accumulation [4].

Neutrophils have been seen as the second line of defense. They migrate out of the pulmonary capillaries and into the air spaces and kill ingested opportunistic microbes (fungi, protozoa, bacteria, viruses) [5].

Lymphocytes are found in both airways and lung parenchyma and are divided into two major populations: thymus-derived T cells and bone marrow-derived B cells. T cells have two major subsets—cluster of differentiation (CD)4+ and CD8+—with further division into Th1 (cellular immunity) and Th2 (humoral immunity). Th2 cytokines include IL-4, IL-5, IL-9, and IL-13, and they promote secretion of immunoglobulin (Ig)E and eosinophilic responses. Therefore, a dysregulated Th1/Th2 response has been linked to a diversity of chronic inflammatory conditions, such as asthma and chronic bronchitis [6]. Th17 is a third subset of Th cells, which are characterized by their production of IL-17. They also play an important role in host defense and several inflammatory disorders [7].

Although inflammatory cells are essential in modulating acute and chronic airway inflammation, epithelial, endothelial and mesenchymal cells also participate in the inflammatory process [8,9]. Recent genetic, structural and functional studies have identified the respiratory epithelium as a main contributor in the development of inflammatory airway disorders [10]. The airway epithelium functions by responding to environmental stimuli, not only via leukocyte recruitment, but also through secretion of chemokines, cytokines, and antimicrobial peptides [11,12,13,14]. The bronchial mucosa consists of a muco-ciliated, pseudostratified epithelium with predominantly ciliated cells, as well as mucus-secreting goblet cells (5–15%) in the large airways and basal cells/club cells in the smaller airways [15,16,17]. It represents a physical and chemical barrier by: (1) apical junctions lining the upper and lower airways, (2) mucociliary clearance, and (3) secretion of mediators regulating inflammation, chemotaxis, and antimicrobial defense as mentioned above [18,19,20,21,22]. In addition, airway epithelial cells also initiate and regulate the innate and adaptive immunity of the lung via pattern-recognition receptors and trans-epithelial immunoglobulin transport [23]. In chronic inflammatory airway diseases, epithelial dysfunction is one of the key features leading to altered epithelial integrity and disrupted physical, chemical, and immune barrier functions [23].

## 2. Mediators of Chronic Inflammatory Airway Diseases

Cytokines are polypeptides and secreted by various cell types, including inflammatory cells and the airway epithelium [24]. They have autocrine, paracrine, or endocrine functions to regulate immunity and inflammation. Chemokines are cytokines that serve as chemotactic agents or chemoattractants for other cells. Furthermore, there is redundancy in cytokine-induced effects with different cytokines acting either synergistically or antagonistically. Interleukins are a subgroup of cytokines and can be classified as pro- or anti-inflammatory. Key pro-inflammatory interleukins are IL-1β, IL-6, and IL-8; they can activate the immune system and contribute to the acute inflammatory response by promoting antigen presentation, inflammatory cell activation, and expression of enzymes involved in matrix degradation [25]. Important anti-inflammatory interleukins include IL-1ra and IL-10, which can be secreted by alveolar macrophages and attenuate lung inflammation [26,27]. IL-10 can also inhibit the production of pro-inflammatory cytokines by activation of T (thymus) cells and monocytes [28].

There is increasing evidence for cytokine dysregulation in many chronic pulmonary diseases. In allergic asthma, IL-4 and IL-13 are produced by antigen-presenting cells, which lead to IgE production by B (bone marrow) cells and subsequent mast cell degranulation. In addition, eosinophils are stimulated by IL-5 that is produced by Th2 cells [29]. The airway epithelium can also produce specific cytokines favoring Th2 and/or Th17 cell differentiation, such as IL-33 promoting IL-5 production and eosinophilia [30]. Altogether, these secreted cytokines can initiate an asthmatic attack.

Many cytokines, including IL-1β and IL-6, have been shown to have a major role in COPD [31]. Additionally, IL-1β levels were shown to be increased in the acute respiratory distress syndrome [1,32].

The following paragraphs will focus on interleukins and their roles in three major chronic inflammatory airway diseases including COPD, bronchiectasis, and allergic asthma.

### 2.1. COPD

COPD is currently the third leading cause of global mortality, according to the World Health Organization (WHO), and results in more than 120,000 deaths each year. Cigarette smoke is responsible for the majority of cases [33]. COPD is characterized by progressive airway inflammation and acute exacerbations (AECOPD) due to infections, which are the major causes that lead to a significant increase in mortality. Chronic inflammation of the airways has been shown to play a key role in the pathophysiology of both COPD and AECOPD, which is associated with increased numbers of goblet cells, mucus gland hyperplasia, fibrosis and pulmonary emphysema [34]. In this environment, activated macrophages release inflammatory mediators and chemotactic factors, such as tumor necrosis factor (TNF)-α, IL-6, IL-8, monocyte chemotactic peptide (MCP)-1, leukotriene (LT) B-4, and reactive oxygen species, which further promote airway destruction. Under the influence of IL-8 and leukotriene (LT)B-4 in particular, neutrophils migrate to the respiratory tract and cause mucus hypersecretion by submucosal glands and goblet cells [35,36,37,38]. Dendritic cells have also been shown to play a key role in activating a variety of inflammatory and immune cells including macrophages, B and T lymphocytes, and neutrophils in COPD [39,40,41].

The respiratory epithelium has been shown to secrete IL-1β and IL-8, which contribute to the development of small airways fibrosis [42,43,44]. Furthermore, IL-4 and -13 levels were shown to be elevated in central airways of smokers with chronic bronchitis, when compared to those of asymptomatic smokers [45]. However, Imaoka and coauthors assessed serum levels of IL-4 and IL-13 in lungs of patients with severe COPD, smokers, and nonsmokers and could not find significant differences among these groups [46].

IL-18, a pro-inflammatory cytokine, has been shown to be produced intracellularly by caspase-1 cleavage from a biologically inactivated precursor, pro-IL-18, and secreted by activated macrophages [47]. IL-18 proteins are expressed in the lungs of healthy individuals at low levels [48] serving as a co-factor for both Th1 and Th2 cell development [49,50,51]. Serum levels of IL-18 in COPD patients and smokers have been demonstrated to be elevated and negatively associated with the predicted forced expiratory volume in one second (FEV1), when compared to nonsmokers. The highest serum levels were found in patients with COPD stages III and IV according to the Global Initiative for Chronic Obstructive Lung Disease (GOLD) [46]. In summary, these studies demonstrate that expression and secretion of various interleukins are altered in COPD, but further studies are needed to define their pathophysiological roles.

### 2.2. Bronchiectasis

Bronchiectasis is an incurable pulmonary disorder, which is either a local or diffuse process affecting the bronchi [52]. Sometimes described as a disease, bronchiectasis can be more appropriately thought of as a terminal pathological result of continuous airway inflammation, recurrent infection, and bronchial wall destruction, due to various primary causes [53,54]. Bronchiectasis was always thought to be a rare disorder but recent reports suggest that bronchiectasis is much more common, with an estimation of 139 cases per 100,000 persons, and its incidence and prevalence are rising within the aging population [55]. Numerous causes can lead to the development of bronchiectasis, with post-infection bronchiectasis being the most common [56]. Among the genetic diseases, cystic fibrosis (CF) is the most common cause; therefore, bronchiectasis has been divided into two groups: CF and non-CF bronchiectasis.

Controlled inflammation is essential for fighting infection; however, when in excess, it will lead to destruction of the lung and host cells [57]. The bronchiectatic airway epithelium can produce an exaggerated pro-inflammatory cytokine response due to infectious stimuli [58]. Therefore, the characterization of relevant inflammatory mediators is important for the development of more specific therapeutic approaches to control inflammation and improve health outcomes in individuals suffering from bronchiectasis [59].

CF has been used as a model disease to study the pathomechanisms leading to bronchiectasis. Interestingly, the CF airways are considered normal at birth, with the exception of some plugging of submucosal gland ducts [60]. Since affected infants already show signs of inflammation and bacterial infection, especially with Staphylococcus aureus soon after diagnosis, lung disease begins early in life [61]. Infants with normal lung function and no apparent bacterial colonization have already-high concentrations of inflammatory markers, such as interleukin 8 (IL-8), peroxidases, and their oxidants in bronchoalveolar lavage (BAL) fluid [61]. The presence of respiratory symptoms has also been associated with elevated neutrophil count despite no evidence of infection [62]. These findings have been controversial, since others suggest infection preceding inflammation in infants with CF [63]. In addition, neutrophils are key inflammatory mediator cells in CF airways and contribute to tissue damage and disease progression. They secrete numerous different mediators that are critical for the neutrophil immune response. Unfortunately, a significant amount of those mediators leak from neutrophils during apoptosis and phagocytosis leading to further airway destruction [64].

### 2.3. Allergic Asthma

Allergic asthma is a chronic inflammatory airway disease, which occurs in response to inhaled allergens [65]. It is characterized by reversible airway obstruction with airway hyperresponsiveness, submucosal infiltration of eosinophils and Th2 cells, mucus hypersecretion, and airway remodeling [66]. Allergic asthma is a type 1 hypersensitivity reaction, which includes allergen-specific IgE molecules cross-linking to high-affinity Fcε receptors of basophils and mast cells. This leads to an immediate release of leukotrienes, prostaglandins, and histamines that induce contraction of airway smooth muscle cells, edema and mucus secretion leading to bronchoconstriction. Furthermore, locally produced chemokines recruit eosinophils, macrophages, neutrophils, and T lymphocytes [66]. Cytokines are also released by eosinophils and epithelial cells, which ultimately initiate a chronic airway remodeling process, further recruitment of eosinophils, and stimulation and continued production of IgE by B cells [66,67]. IL-4, IL-5, IL-6, and IL-13 have been shown to maintain this cycle of allergic inflammation [68] with IL-4 promoting Ig isotype switching [69], IL-5 inducing eosinophil differentiation and migration [70], and IL-13 causing goblet cell hyperplasia and airway hyperresponsiveness [71,72]. IL-33 is another pro-inflammatory cytokine, which has been shown to drive neutrophil inflammation and promote airway remodeling in asthma [73,74].

### 2.4. Current Therapeutic Strategies and Their Effect on Airway Inflammation

With chronic airway inflammation and remodeling serving as key factors for the severity of asthma, COPD, and bronchiectasis, three classes of inhaled therapies have been utilized to aid in symptom management. Inhaled corticosteroids (ICSs) are commonly used in asthma to suppress airway inflammation and consequently reduce airway hyperresponsiveness [75]. ICSs can often be combined with long acting β adrenergic receptor (B_2_) agonists (LABA) to help relieve symptoms of severe asthma [76]. LABA and long acting muscarinic antagonists (LAMA) are bronchodilators commonly used for the symptomatic treatment of COPD. As a step-up therapy, LABAs and LAMAs are often given as a dual therapy in COPD patients to reduce bronchoconstriction, mucus secretion, and AECOPD [77,78]. The triple inhaled therapy is given to COPD patients who have continuing exacerbations or patients with severe persistent asthma. Although these inhaled therapies have been useful for their management of chronic airway inflammation, they also have their shortcomings. ICSs have high efficiency in treating asthma but are only partially effective in controlling airway inflammation. Moreover, ICSs have been linked to adverse cardiovascular effects in COPD patients, growth stunts in pediatric CF bronchiectasis patients, and withdrawal complications [75,79,80]. The biggest limitation of these inhaled therapies is their inability to aid in regulating disease modification and/or progression.

Regarding other anti-inflammatory therapy options, patients with CF bronchiectasis can use high dose ibuprofen. This is a recommended anti-inflammatory treatment, but mainly used in the pediatric population due to gastrointestinal side effects [76,78,80]. Azithromycin, an antibiotic with anti-inflammatory properties, has been shown to reduce exacerbation frequency in COPD patients when taken daily for one year [81]. A similar effect of azithromycin use included less exacerbations, fewer positive cultures, less needed oral antibiotics, and significant improvements in lung function in CF patients, when taken chronically for at least six months [82]. For COPD, phosphodiesterase 4 (PDE4) inhibitors have also emerged as an anti-inflammatory treatment option. A recent meta-analysis study showed that PDE4 inhibitors improved lung function and reduced AECOPD frequency, but they had little effect on quality of life or symptoms [83]. Patients on this therapy frequently experienced gastrointestinal adverse effects and weight loss as well psychiatric adverse events. Therefore, current COPD treatment guidelines only support their use as an add-on therapy in patients with persistent symptoms or exacerbations despite optimal COPD management [83]. Finally, theophylline (dimethylxanthine), one of the oldest medications, has been used to treat airway diseases. It is a bronchodilator but also shows anti-inflammatory effects via inhibition of PDE4 and histone deacetylase-2 activation [84]. Its use, however, has decreased over the years mainly due to the high frequency of adverse effects even when used at conventional doses, such as headaches, nausea and vomiting, but also convulsions and cardiac arrhythmias [85].

Since COPD, CF, and non-CF bronchiectasis are associated with increased mucus secretion and impaired mucociliary clearance, mucus clearance therapies have been indicated and have shown improvement in both symptoms and chronic airway inflammation [86,87]. These therapies include not only a variety of airway clearance therapies (ACT) [88] but also the introduction of mucolytics, such as dornase alfa, hypertonic saline, and inhaled antibiotics. Airway clearance therapies are numerous but, so far, none have been shown to be superior. Therefore, the current recommendation is to perform ACT on a regular basis in all CF patients [88]. Dornase alfa is a recombinant human deoxyribonuclease and has shown to facilitate mucus clearance in the lung, thereby preventing airway obstruction, exaggerated inflammation and, ultimately, lung function decline [89]. Similarly, hyperosmolar saline has been used as an effective expectorant in patients with CF bronchiectasis. Current evidence only supports the use of hypertonic saline and dornase alfa in patients with CF [90]. In summary, current established anti-inflammatory treatments in these chronic airway diseases are mainly symptomatic and broad in targeting multiple pro-inflammatory signaling pathways; therefore, their use is limited by various side effects.

## 3. Current Cytokine Specific Treatment Strategies

With the frequency and severity of airway inflammation in chronic lung disorders, a growing area of interest has been centered on the identification of factors that drive disease, including cytokines/chemokines, and their potential as viable therapeutic targets.

Th2 cytokines are the primary targets for biologic therapeutics for chronic airway diseases associated with eosinophilic inflammation. So far, anti-IL-5 therapy in asthma has been approved and cleared by the Food and Drug Administration (FDA), and clinical trials have been performed since 2000 with the first inhibitor, mepolizumab, which was approved for its use in 2015. Mepolizumab was shown to promote a reduction in eosinophils, acute exacerbations, and the need for steroids [91,92]. In recent years, two additional anti-IL-5 antibodies (reslizumab and benralizumab) have been approved by the FDA for their safety and efficacy in reducing asthma exacerbations [93,94]. IL-5 therapeutics are currently being studied for an indication in COPD; phase 3 trials are currently being conducted using benralizumab and mepolizumab. The first results showed that benralizumab displayed a mild beneficial effect on lung function, while treatment with mepolizumab reduced COPD exacerbations [95,96].

Similar to the IL-5 treatment strategies in COPD, an anti-IL-4 antibody has been studied in a clinical trial as an indication for asthma, and the IL-4 receptor α inhibitor, dupilumab, has shown notable improvements in exacerbations and lung function from asthmatic patients that are already on dual therapy with ICSs and LABA [97,98]. Recently, dupilumab was approved by the FDA for its asthma indication but has not been examined for its efficacy in COPD to date. The anti-IL-13 therapeutics lebrikizumab and tralokinumab were used in phase 3 clinical asthma trials; however, the inhibitors showed negligible to no effects on lung function or exacerbation reduction [99].

## 4. Future Therapeutic Strategies

Several pro-inflammatory cytokines have been shown to be upregulated and characterized as contributors to disease severity in asthma, COPD, and CF bronchiectasis making them appropriate therapeutic targets. These will be discussed in detail in the following paragraphs.

### 4.1. Inhibition of IL-1β as Therapeutic Strategy

IL-1β is a pro-inflammatory cytokine with increased expression in asthma, COPD, and CF bronchiectasis. Our group has demonstrated that the bronchial epithelium secretes IL-1β in response to cigarette smoke [12] leading to lung inflammation, emphysema, fibrosis, and goblet cell hyperplasia in mice [100]. Pro-IL-1β, produced by activation of the nuclear factor (NF)-κB signaling cascade, is converted into its cytosolic active form by the inflammasome, which mediates the auto-activation of caspase-1, cleaving pro-IL-1β into its biologically active form [101,102].

Infection with *Pseudomonas aeruginosa* (PsA), one of the most clinically relevant pathogens in CF bronchiectasis, can lead to an increase in levels of IL-1β in BAL fluid from these patients [103,104]. In addition, polymorphisms in the *IL1B* gene have been shown to be associated with disease severity [105]. Along these findings, Muselet-Charlier and coauthors found a rapid IL-1β mediated activation of NF-κB in a CF lung epithelial cell line [106]. CF mice exhibited augmented IL-1β signaling in response to PsA, and PsA-mediated lung inflammation and bacterial load were attenuated by a neutralizing IL-1β antibody [107]. In addition, dysfunction of the inflammasome, namely pyrin domain containing 3 (NLRP3) as a key activating factor, led to IL-1β-dependent inflammation in both murine and human CF bronchiectasis disease. This NLRP3 activity was shown to be regulated by IL-1 receptor antagonist (IL-1RA) in a negative feedback loop, thereby providing a potential therapeutic angle to attenuate CF airway disease by chronic colonization [108].

Altogether, these data highlight the involvement of IL-1β in smoke and CF-related inflammatory airway disease and IL-1β inhibition as potential future therapeutic application.

IL-1β has also been shown to be upregulated in neutrophilic asthma compared to eosinophilic and pauci-granulocytic asthma [109]. He et al. conducted a meta-analysis summarizing 15 case-control studies and analyzed the association between asthma risk and genetic polymorphisms in IL-1β -511C/T and IL-1RA. No association was found for the IL-1β -511C/T polymorphism, but the IL-1RA polymorphism was related to an increased risk of asthma, which was independent of ethnicity and age [110]. Furthermore, Besnard et al. concluded that inflammasome-induced IL-1β production ultimately contributes to the control of allergic asthma by enhancing Th17 cell differentiation [111]. Another study along these lines could demonstrate that the IL-1 receptor antagonist and IL-1 type-II receptor attenuated both IL-5- and IgE-mediated changes in airway smooth muscle cell responsiveness. Human airway smooth muscle cells, exposed to IL-5, IL-1β and IgE, upregulated expression levels of both stimulatory and inhibitory IL-1 axis molecules, which suggests that modulation of the interleukin-1 axis may potentially also have significant therapeutic implications in the treatment of asthma [112].

So far, small clinical trials have been performed examining the role of IL-β blockade for asthma and COPD. Canakinumab is a high-affinity human immunoglobulin G kappa (IgGk) monoclonal antibody that targets Il-1β by neutralizing its bioactivity. One randomized double-blinded trial in asthmatic patients has been conducted so far, which consisted of two single administrations on day 1 and day 15 in patients with mild asthma. Patients were allowed to stay on other anti-asthmatic drugs and allergen challenge was performed on day 0 and day 28. The results showed that canakinumab led to a 28% decrease in the late asthmatic response. Furthermore, a single dose of canakinumab significantly reduced circulating IL-1β levels for the time measured. Although this trial was small and included only 16 patients, the results were positive and promising [113].

The impact of canakinumab on pulmonary function in COPD was also assessed in a phase 1/2 study, which included 147 participants. Individuals received either drug or placebo intravenous infusion at weeks 1, 5, 7, and thereafter every 4 weeks for a total of 45 weeks. The primary outcome measure did not show any significant difference in lung function between groups. Is this study alone sufficient to “disqualify” canakinumab, or were the studied outcome measures just not sensitive enough? Should the study have been conducted for a longer time and should COPD stages, progression, or COPD-associated inflammation have been assessed instead? These are all valid questions and may have contributed to a different outcome; therefore, this study alone should not preclude the use of canakinumab as a potential future therapy in COPD.

Anakinra is a recombinant IL-1ra protein that can block IL-1β mediated effects and therefore, represents an attractive novel therapy for chronic inflammatory airway diseases. Hernandez et al. conducted a small study to assess the effect of anakinra on the acute neutrophil response after an inhaled endotoxin challenge in 17 healthy volunteers. The authors could show that anakinra effectively reduced neutrophilic airway inflammation without any serious adverse effects, thus making anakinra a potential target for the treatment of asthma with neutrophil predominance [114]. A follow up phase 1/2 trial is currently enrolling patients with mild allergic asthma to test the efficacy of anakinra and its therapeutic role in allergic asthma. Furthermore, both anakinra and canakinumab could have potential therapeutic anti-inflammatory implications in bronchiectatic lung diseases. Recent reports have identified the IL-1 receptor for its critical role in the pathogenesis of a murine CF model, in which anakinra was able to efficiently block CF-related airway inflammation and mucus obstruction [115].

Nevertheless, caution should be taken when using IL-1 inhibitory therapy due to the primary role of IL-1β activating the inflammasome and protecting against respiratory infections. IL-1 inhibition may render patients with chronic inflammatory respiratory disorders more susceptible to active airway infection and exacerbations. Therefore, targeted approaches to suppress the inflammatory response need to be assessed thoroughly and used wisely for certain subgroups of patients.

### 4.2. IL-6 Blocking Antibody Therapy

IL-6 can be produced by both inflammatory and primary lung epithelial cells in response to a variety of different stimuli [116,117,118]. The age-dependent onset of frailty, loss of lung function, and increased prevalence of COPD suggests involvement of aging related mechanisms or “inflammaging”. One concept characterizes aging as a proinflammatory condition due to the underlying immune dysfunction with COPD. Therefore, emphysema development has been shown to represent an “accelerated aging phenotype” [119,120]. Aging-related pro-inflammatory cytokines, especially IL-6, have been linked to negative health outcomes and mortality, and speculated to result in persistent, low grade activation of chronic inflammation [121]. Other explanations for this aging-associated rise in circulating IL-6 levels include increased oxidative stress or persistent viral infections (herpes or cytomegalovirus) as observed in the frail and elderly [122,123]. Characterizing the mechanism of IL-6 in affecting frailty, lung disease (specifically COPD), and adverse outcomes may prove to be an important field of future research. In CF bronchiectasis, Nixon and co-authors demonstrated that IL-6 sputum levels were negatively associated with FEV1 and forced vital capacity (FVC) with IL-6 decreasing after antibiotic treatment [124].

Analyzing the role of IL-6 in asthma revealed that IL-6 mRNA was constitutively present in mouse primary lung epithelial cells, but not in resident immune cells. Interestingly, IL-6-induced IL-4 production during Th2 differentiation has been shown to inhibit Th1 differentiation [125] and, together with transforming growth factor (TGF)-β, IL-6 has also been documented to promote Th17 cell differentiation [126,127]. Asthmatic patients (including allergic and intrinsic asthma) were shown to have increased IL-6 serum and BAL fluid levels [118,128,129]. Two studies showed that the levels of IL-6 in sputum of asthmatic patients inversely correlated with FEV1 [130,131]. This also held true in obese asthma patients [132].

These studies indicate that IL-6 seems to be directly involved in both the pathogenesis of asthma as well as its associated progressive loss of lung function.

Blocking antibodies to IL-6 and its receptor have been studied in other inflammatory diseases, such as rheumatoid arthritis and Crohn’s disease. Clinical trials for tocilizumab, a blocking antibody for the IL-6 receptor, revealed decreased IL-6 levels, improved disease activity scores, and symptoms in rheumatoid arthritis. The antibody was approved in 2010 but has not been tested in chronic inflammatory airway diseases to date [133]. Therefore, targeting IL-6 could be a potential novel and promising treatment strategy to suppress not only chronic airway but also its associated systemic inflammation, COPD-associated frailty and decline in lung function.

### 4.3. Inhibition of IL-8 Signaling as an Anti-Inflammatory Therapy Approach

IL-8 is an essential cytokine in neutrophil recruitment and the inflamed bronchial epithelium is a major source of IL-8 [134,135]. We have shown that increases in TGF-β and fibroblast growth factor (FGF) 23 led to increased bronchial epithelial IL-8 secretion [13]. In addition, NF-κb has been shown to mediate IL-8 expression in CF bronchiectasis with an upregulation in early phases of PsA colonization [106,136,137]. IL-1β also induced IL-8 in the bronchial epithelium of CF patients [101,138]. Other reports, however, demonstrated no differences in IL-8 secretion between CF and control airway epithelial cell cultures, but increased IL-8 secretion was observed after infection with PsA [139].

IL-8 has also been characterized as a key contributor to the development of COPD; the COPD bronchial epithelium has been shown to have a higher baseline expression of IL-8, thus leading directly to mucus hypersecretion by induction of the mucin genes *MUC5AC* and *MUC5B* [140,141,142]. Both cigarette smoke and tumor necrosis factor (TNF)-α have been shown to synergistically stimulate macrophage IL-8 production [143]. This suggests that cigarette smoke is a sufficient stimulator for excessive IL-8 release in COPD airways [144]. Furthermore, neutrophils and circulating IL-8 have been shown to accumulate in AECOPD [145,146], with IL-8 being negatively associated with lung function [147].

One study in asthmatic patients assessed sputum concentrations of IL-8, demonstrating increased levels in severe versus mild asthma [148]. Another report showed that IL-8 in tracheal aspirates was significantly higher in patients who were intubated for acute severe asthma, when compared to control individuals without lung disease [149]. This finding was consistent with a previous report demonstrating a marked increase in IL-8 in BAL fluid from patients with status asthmaticus [150]. Hosoki et al. identified neutrophils and IL-8 as the only inflammatory components in BAL fluids that distinguished controlled asthma from uncontrolled asthma with an inverse correlation to FEV1 [151]. In summary, these studies suggest that IL-8 is an essential pro-inflammatory mediator in asthma and a possible prognostic biomarker for disease severity.

So far, the effects of blocking IL-8 have been examined in COPD; however, no significant clinical improvements have been detected in patients in these small trials [152]. Interestingly, the chemokine receptor C-X-C chemokine receptor (CXCR) 2 is a chemo attractant for neutrophils and has been shown to aid in the production of IL-8. Its antagonist has been shown to decrease the levels of IL-8 production making it a clinical target. Treatment with the CXCR2 receptor antagonist, navarinxin, has shown a significant reduction of sputum and blood neutrophils in asthmatic patients without any effect on lung function [153]. In clinical COPD trials, navarinxin caused small improvements in lung function [154]. An additional CXCR2 antagonist, danirixin, is currently under investigation for clinical usage [76]. SB-656933, a receptor antagonist for CXCR2, has been tested in patients with CF bronchiectasis and was shown to be well-tolerated (except minor reports of headaches) with improvements in sputum neutrophils but no improvement in symptoms or lung function [155]. Therefore, these therapies are promising but will need further evaluation and testing.

**Figure 1:**
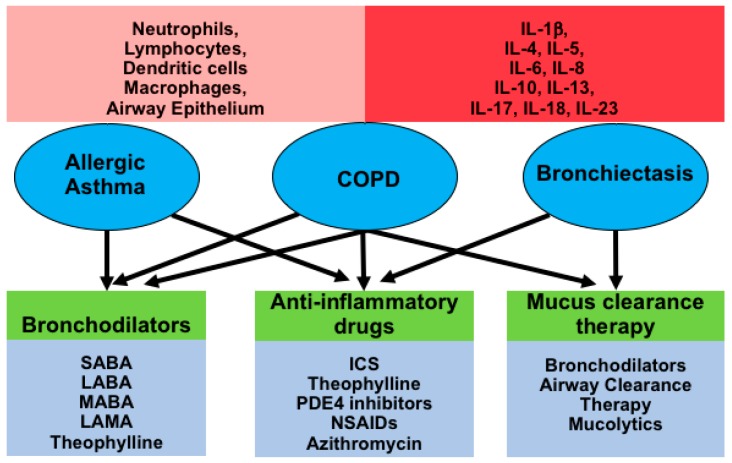
Diagram depicting inflammatory airway diseases in circles (allergic asthma, COPD and bronchiectasis), which all show airway inflammation with involvement of various cell types and the main cytokines (red rectangles), and current common therapies (green/blue rectangles) used for their treatment. Abbreviations: SABA, short-acting β_2_-adrenoreceptor agonists; LABA, long-acting β_2_-adrenoreceptor agonists; MABA, dual muscarinic antagonist-β_2_-agonist compounds; PDE4, phosphodiesterase 4; SAMA, short-acting muscarinic receptor antagonists, LAMA, long-acting muscarinic receptor antagonists; ICS, inhaled corticosteroids; NSAIDs, nonsteroidal anti-inflammatory drugs.

The diagram below is summarizing the three chronic inflammatory airway diseases discussed in this review and the involvement of different cell types, pro- and anti-inflammatory cytokines and current treatment strategies (Figure 1). 

### 4.4. Future Approaches Targeting IL-17 and IL-23

IL-17 plays an essential role in the pulmonary host defense by inducing neutrophil local release of neutrophil-mobilizing factors, including CXC chemokines [156]. Recent reports are controversial regarding the role of IL-17 in the development of COPD [157,158,159,160]; IL-17 levels were shown to be low in the sputum of individuals with COPD [161], and there was no difference in IL-17 receptor expression levels between individuals with COPD and non-COPD lung disease [162]. However, IL-17 was also shown to activate neutrophils, thereby enhancing epithelial production of IL-8 and MUC5AC synthesis in the bronchial epithelium [163,164]. Taking these results together, IL-17 is most likely part of the protective immunity in COPD.

In bronchiectatic airways, IL-17 was detectable in bronchoalveolar lavage fluid and led to increased expression of MUC5AC and MUC5B. Treatment of epithelial cultures with IL-17 caused an increase of IL-8, thereby contributing to airway inflammation [165]. Hsu and coauthors showed that mice, infected with PsA, had a significant upregulation of IL-17 that was associated with a persistent inflammatory response. A similar response could be mimicked by direct intratracheal administration of IL-17 with attenuation by neutralization of IL-17 [166].

Similarly to the COPD data, Th17 cells and their corresponding cytokines, IL-17A and IL-17F, appeared to contribute to the pathogenesis of primarily severe and steroid-resistant asthma [167]. The asthmatic airway epithelium also produced specific cytokines favoring Th2 and/or Th17 cell differentiation, such as IL-33-promoting IL-5 production and eosinophilia [30].

Due to the evidence presented here, modulation of IL-17 is an attractive potential therapeutic target in chronic inflammatory airway diseases [167,168,169]. Small clinical trials have examined the effectiveness of IL-17 and its receptor antagonist in asthma and COPD. Unfortunately, no improvements in lung function or disease symptoms have been observed in patients with either disease [170,171]. Risankizumab, an anti-IL-23 antibody, is in the early stages of clinical trials for severe asthma [76]. Although the three cytokines are not always regulated synergistically, regulation of the Th17 cytokine axis (IL-23, IL-17A, and IL-22) might prove to be beneficial for several inflammatory diseases. 

**Figure 2:**
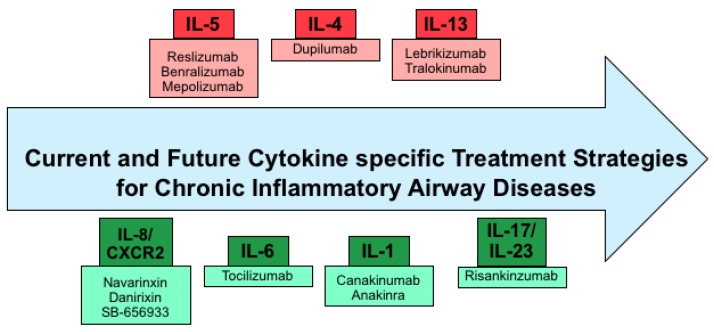
Diagram depicting current and future cytokine specific therapy options, with pink boxes representing established therapies and light green boxes representing therapies that are currently under investigation—cytokines are in either red or green.

Below is a diagram summarizing current and potential future cytokine-targeted therapies in chronic inflammatory airway diseases (Figure 2).

## 5. Conclusions

Managing chronic lung disorders and chronic airway inflammation are global concerns, and their prevalence is increasing due to an aging population and a substantial increase of particulate matter air pollution over recent decades. Developing a better understanding of pro-and anti-inflammatory cytokines that regulate and/or drive disease severity serves as a beneficial tool in the development of modern therapeutics. Even though extensive progress has been made thus far, most of our therapeutic approaches for chronic inflammatory airway diseases target broadly with significant side effects. Recent attempts of specific anti-cytokine therapies have only shown some benefits. Future research is needed to help find novel and more specific targets, or for more in-depth evaluation of the specific therapies we already have. In addition, the right patient subgroups and timing for initiation of treatment need to be thoroughly investigated, since inflammation is needed to activate a proper immune response. This might be the explanation for some of the negative results of current clinical anti-cytokine trials. Therefore, it is worth conducting long-term studies with anakinra and canakinumab, if lung function or disease progression is an outcome measure. Furthermore, other outcome measures might be more appropriate when assessing anti-inflammatory treatments, such as exacerbation or hospitalization frequency. Hopefully, further research will help in the future to not only identify novel treatments for chronic inflammatory lung disorders but also aid in a better understanding of their pathophysiology.
